# Thyroid Function and Perchlorate in Drinking Water: An Evaluation among California Newborns, 1998

**DOI:** 10.1289/ehp.8176

**Published:** 2005-12-15

**Authors:** Patricia A. Buffler, Michael A. Kelsh, Edmund C. Lau, Charlotte H. Edinboro, Julie C. Barnard, George W. Rutherford, Jorge J. Daaboul, Lynn Palmer, Fred W. Lorey

**Affiliations:** 1 University of California, Berkeley, California, USA; 2 Exponent, Inc., Health Practice, Menlo Park, California, USA; 3 University of California, San Francisco, California, USA; 4 Nemours Children’s Clinic, Orlando, Florida, USA; 5 California Department of Health Services, Genetic Disease Branch, Richmond, California, USA

**Keywords:** drinking water, newborn screening, perchlorate, primary congenital hypothyroidism, thyroid-stimulating hormone

## Abstract

Perchlorate (ClO_4_^−^) has been detected in groundwater sources in numerous communities in California and other parts of the United States, raising concerns about potential impacts on health. For California communities where ClO_4_^−^ was tested in 1997 and 1998, we evaluated the prevalence of primary congenital hypothyroidism (PCH) and high thyroid-stimulating hormone (TSH) levels among the 342,257 California newborns screened in 1998. We compared thyroid function results among newborns from 24 communities with average ClO_4_^−^ concentrations in drinking water > 5 μg/L (*n* = 50,326) to newborns from 287 communities with average concentrations ≤5 μg/L (*n* = 291,931). ClO_4_^−^ concentrations obtained from the California Drinking Water Program provided source-specific data for estimating weighted average concentrations in community water. Fifteen cases of PCH from communities with average concentration > 5 μg/L were observed, with 20.4 expected [adjusted prevalence odds ratio (POR) = 0.71; 95% confidence interval (CI), 0.40–1.19]. Although only 36% of all California newborns were screened before 24 hr of age in 1998, nearly 80% of newborns with high TSH were screened before 24 hr of age. Because of the physiologic postnatal surge of TSH, the results for newborns screened before 24 hr were uninformative for assessing an environmental impact. For newborns screened ≥24 hr, the adjusted POR for high TSH was 0.73 (95% CI, 0.40–1.23). All adjusted odds ratios (ORs) were controlled for sex, ethnicity, birth weight, and multiple birth status. Using an assessment of ClO_4_^−^ in drinking water based on available data, we did not observe an association between estimated average ClO_4_^−^ concentrations > 5 μg/L in drinking water supplies and the prevalence of clinically diagnosed PCH or high TSH concentrations.

Perchlorate (ClO_4_^−^) has been used industrially as an oxidizer for rocket fuels and propellants and in explosives and pyrotechnics. In the 1950s and 1960s, it was also used as a treatment for hyperthyroidism associated with Graves disease. Environmental emissions have resulted in detection of ClO_4_^−^ levels greater than 4 ppb (micrograms per liter) in drinking water supplies perhaps affecting over 11 million people in 35 U.S. states (National Research Council 2005). The detection of ClO_4_^−^ in numerous groundwater sources in the United States, including those of many communities in California, has raised concerns about potential health impacts. At therapeutic levels, ClO_4_^−^ competitively inhibits iodide transport, thus reducing the concentration of available iodine for hormone production and subsequent release (Wolff 1998). Specific concerns regarding environmental exposures pertain to potential disruption of the thyroid in the newborn and subsequent neurodevelopmental effects (DiGeorge 1987; Fisher 1996). Hypothyroidism in newborns (congenital hypothyroidism) in iodine-sufficient regions is most commonly caused by developmental defects of the thyroid (thyroid dysgenesis) (Foley 2000). The prevalence of thyroid dysgenesis in iodine-sufficient regions is approximately 1 per 4,000 newborns (Fisher 1996) and has been reported to account for up to 80% of cases of congenital hypothyroidism (Foley 2000).

The hypothalamic–pituitary–thyroid axis operates as a negative feedback loop to provide regulation of thyroxine (T_4_) and thyroid-stimulating hormone (TSH) concentrations and can be affected by changes in environmental conditions, nutrition, and drugs (Reed 2000; Scanlon and Toft 2000). Immediately after birth, a normal surge in TSH concentration occurs, which falls rapidly after the first 24 hr (de Zegher et al. 1994). Early collection (< 24 hr of age) of screening samples will detect this physiologic elevation of TSH and may account for a large portion of false-positive primary congenital hypothyroidism (PCH) results (Allen et al. 1988). Program evaluations of California newborn screening data showed that ethnicity, birth weight, and sex influenced the prevalence of PCH (Waller et al. 2000). These factors may apply to concentrations of T_4_ and TSH as well; however, very few data have been reported to evaluate these associations.

Recent epidemiologic studies examined associations between potential exposure to ClO_4_^−^ from drinking water and newborn thyroid function or PCH prevalence (Brechner et al. 2000; Crump et al. 2000; Kelsh et al. 2003; Lamm and Doemland 1999; Li et al. 2000a, 2000b). It has been proposed that consumption of ClO_4_^−^ water, at any concentration, by a mother during pregnancy may depress thyroid function in her newborn and therefore increase the risk of PCH or other neurodevelopmental outcomes.

Published epidemiologic studies have not shown a consistent association between ClO_4_^−^ in drinking water and congenital hypothyroidism or altered thyroid function. One study of Arizona newborns reported an association between ClO_4_^−^ in drinking water and thyroid function (Brechner et al. 2000), whereas five other studies in California, Nevada, and Chile did not provide evidence supporting this association (Crump et al. 2000; Kelsh et al. 2003; Lamm and Doemland 1999; Li et al. 2000a, 2000b). Interestingly, no association was noted between exposure to ClO_4_^−^ in drinking water and TSH in two studies with more quantitative exposure information (Crump et al. 2000; Li et al. 2000a). Thus, it is possible that the inconsistent results regarding exposure to ClO_4_^−^ in drinking water and TSH concentrations in newborns may be the result of methodologic issues (Kelsh et al. 2003; Lamm 2003). The objectives of this investigation were to assess whether there was epidemiologic evidence of higher rates of PCH or high TSH levels among newborns in California communities with and without detectable ClO_4_^−^ in their drinking water supplies, and to evaluate the extent to which inconsistent results could be the result of the methodologic differences noted (Kelsh et al. 2003; Lamm 2003). This study expanded our earlier investigation of PCH and TSH concentrations in a southern California community where ClO_4_^−^ had been detected (Kelsh et al. 2003). The present study used data from the California Newborn Screening (NBS) Program for 1998 to examine the prevalence of PCH and TSH levels for all California newborns whose mothers resided in communities where water supplies were tested for ClO_4_^−^ in 1997 and 1998.

Until late 1997, California used a two-tiered T_4_-TSH screening program to screen for PCH. In this program, all newborns were screened for T_4_, and only those with a low T_4_ (e.g., < 10 μg/dL) had their TSH measured. Beginning in December 1997, the California Department of Health Services (DHS) NBS Program replaced the two-stage screening procedures with TSH-only testing for PCH. Thus, since late 1997, TSH has been measured for all California newborns tested under the new program. TSH concentration is considered a more stable biomarker for the evaluation of potential PCH than T_4_ (DiGeorge 1987; Fisher 1996). According to the American Academy of Pediatrics (1993), the ideal time to collect blood from newborns for TSH screening is between 2 and 6 days of age.

This research was reviewed and approved by the Committee for the Protection of Human Subjects of DHS for the use of NBS Program data. A subset of the genetic screening data relevant to hypothyroidism and TSH (excluding personal identifying information) was made available to the research team by the Genetics Disease Branch (GDB) of DHS, and the data analyses were conducted in collaboration with investigators of the GDB of DHS (FWL and LP).

## Materials and Methods

### Study population.

The study population consisted of all California newborns screened by the California NBS Program in 1998 whose mothers resided in communities where groundwater drinking sources were tested for ClO_4_^−^ by the California Drinking Water Program (DWP). Newborns in this study were classified into ClO_4_^−^ exposure groups based on the average ClO_4_^−^ concentrations calculated for the mother’s city of residence. The ClO_4_^−^ water-testing data that corresponded to the available NBS Program data (birthdates January 1998–December 1998) were the 1997 and 1998 DWP testing data. There were nearly 800 water sources from approximately 150 different water systems tested for ClO_4_^−^ in the 1997–1998 period, representing approximately 200 California communities. Cities and towns served by water systems that were not tested for ClO_4_^−^ in 1997 or 1998 were not included in this analysis. This resulted in the exclusion of 166,894 of 509,151 (32.8%) 1998 newborns for whom exposure data were unavailable. To address the potential impact of the Colorado River as a source of ClO_4_^−^, we conducted a subgroup analysis that included the 102,966 newborns from communities that did not receive Colorado River water and where groundwater was tested for ClO_4_^−^ and the 239,291 newborns who lived in areas that received Colorado River water. Colorado River water has had ClO_4_^−^ contamination at various concentrations depending on where measurements were taken. The concentration at the point where water was diverted for use in southern California ranged from 5.0 to 9.0 μg/L (U.S. EPA 1999). Colorado River water was then mixed with other sources before delivery to consumers. The proportion of Colorado River water in southern California drinking water varied considerably depending upon the city and time of year.

### Study variables.

Information abstracted from the NBS Program records for 1998 was used to construct variables for this analysis. Some missing or erroneous values were imputed or corrected. Details of the data editing and management process have been described elsewhere (Kelsh et al. 2003). The health or biomarkers outcomes investigated were diagnosis of PCH and high TSH level (defined by GDB as > 25 μU/mL). Elevated TSH is a biomarker for thyroid function, whereas PCH is a specific clinical end point. The newborn’s physician is responsible for reporting confirmed diagnosis of PCH to the screening program. Once physicians are alerted to a newborn with high TSH concentration, diagnosis of PCH is generally based on confirmatory tests of serum free T_4_ and TSH levels and a detailed physical examination and neonatal history. Assessment may also include thyroid scanning and testing of serum-binding proteins and serum triiodothyronine levels.

Covariate data available from the newborn screening records included age (in hours of life) at time of specimen collection, sex, race/ethnicity, birth weight, and multiple birth status. The exposure variable derived from the California DWP was average ClO_4_^−^ concentration categorized as > 5 μg/L and ≤5 μg/L. In addition, a Colorado River indicator variable was assigned to each record if the mother’s residence received drinking water from the Colorado River.

### California DWP.

The DWP of the California DHS was established to monitor water sources of public water systems. Approximately 80 chemical and six radiological contaminants for which maximum contaminant levels (MCLs) have been established are monitored (California DHS 2003a). The DWP also monitors concentrations of other chemicals for which no MCL has been established. In 1997 and 1998, ClO_4_^−^ was one of the unregulated chemicals monitored by DHS. The DWP initiated ClO_4_^−^ testing of drinking water wells in February 1997 (California DHS 2003b). These data are organized by water system (water company, distributor, or private entity such as a mobile home park); data for each water system may include testing data from multiple sources or wells, and each source may have been tested on multiple occasions.

ClO_4_^−^ exposure estimates were based on samples from wells tested for ClO_4_^−^ from February 1997 through December 1998. We selected 1997 and 1998 water data, assuming these years would span the gestation periods for the 1998 newborns. Because testing data after 1998 may not accurately characterize concentrations in water sources in the 1997 and 1998 period, post-1998 ClO_4_^−^ testing data were not used. Analysis of post-1998 water quality data showed that about 90% of the groundwater sources tested in 1997–1998 were tested again sometime after 1998 and before March 2003. The median number of additional tests performed after 1998 was four (range 1–202). The concentrations in 1997–1998 and subsequent tests were below the nominal detection limit of 5 μg/L for 79% of the water sources with post-1998 testing. Of the remaining sources tested, 15% showed no statistically significant difference in ClO_4_^−^ concentration from samples tested in the two time periods, 3.4% showed a significant increase, and 2.6% had a significant decrease in ClO_4_^−^ concentration. Thus, most ground-water sources remained at below-detection concentration level or showed no significant change in concentration after 1998.

DWP measurements for ClO_4_^−^ concentrations below the test detection limits were recorded inconsistently. Sample concentrations where ClO_4_^−^ was not detected were recorded either as 0.0, < 4, or < 5 μg/L. Most measurements that yielded concentrations too low to quantify were recorded as < 4 or < 5 μg/L based on the lower detection limit at the time of 4–5 μg/L. From 1997 and 1998, 48 water systems had one or more samples with detectable ClO_4_^−^, and 151 water systems had no detectable ClO_4_^−^ in any of their samples. We selected the cutoff point of 5 μg/L to represent concentrations at or below 5 μg/L.

Information for cities or towns served by each water system tested by the DWP was collected either from the DWP file or through Internet searches or telephone interviews conducted by research staff. In addition to relying on local groundwater wells, water systems frequently purchase water from other distributors and wholesalers. Many southern California communities also receive a portion of their water from the Metropolitan Water District (MWD) of Southern California. The MWD distributes northern California and Colorado River surface water to 26 water systems that provide drinking water to nearly 18 million southern California residents (MWD 2003a). Information on Colorado River water allocations was collected through Internet searches and telephone inquiries to major southern California water wholesalers and the Colorado River Water Users Association (Colorado River Water Users Association 2003; MWD 2003b). This information was used to determine whether each city received Colorado River water.

Six samples from four MWD sources were tested in 1997 and 1998. The highest ClO_4_^−^ concentration for these years was 9.0 μg/L, with an average of 4.1 μg/L. No public data source was available to identify the amount of water provided by the MWD to its affiliated water systems. Thus, after blending and mixing with other sources, the proportion of MWD-supplied water reaching consumers in a particular community at a particular time could not be determined.

In the absence of detailed and complete information on water distribution practices for each California water system, the following methods were adopted to estimate average ClO_4_^−^ concentrations for each water system and city. The average water system ClO_4_^−^ concentration was the arithmetic mean of median concentrations from each contributing water source or well. The average ClO_4_^−^ concentration for a given city was then calculated as the weighted average of concentrations from the different water systems that provided drinking water to the city, weighted by the number of water sources, counting the MWD as one source. Thus, the ClO_4_^−^ concentration for a community was estimated as


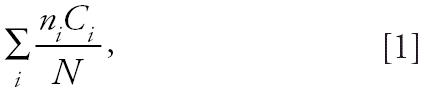


where *n_i_* is the number of drinking water sources contributed by the *i*th water system, *C_i_* is the ClO_4_^−^ average concentration for the *i*th water system, and *N* is the total number of sources from all contributing water systems.

### Statistical analysis.

#### Primary congenital hypothyroidism.

We examined the relationship between PCH occurrence and potential ClO_4_^−^ exposure using logistic regression models to estimate adjusted prevalence odds ratios (PORs). In addition to the drinking water ClO_4_^−^ classification of ≤5 μg/L or > 5 μg/L, covariates in the logistic regression analyses for PCH included birth weight, ethnicity, sex, and indication of multiple births. Only the first newborn in a multiple birth was retained for analysis, as characteristics of the subsequent newborns were unlikely to be independent.

#### Thyroid-stimulating hormone.

The potential relationship between high TSH level and residence in communities with detectable ClO_4_^−^ in drinking water was examined using logistic regression models to estimate adjusted PORs. The continuous TSH variable was dichotomized into “high” or “normal” based on the 1998 DHS GDB cutoff value of 25 μU/mL.

Covariates were those used in the PCH analyses as well as age at time of blood sample collection. Given the documented physiologic postnatal surge of TSH before 24 hr of age, we restricted the TSH analyses to those newborns with an age at specimen collection ≥24 hr. In our analyses, we also examined the subgroup of newborns with normal birth weights. Two-way interactions were examined and found to have minimal effect on the POR and thus were not included in the final model.

## Results

### Newborn screening data.

Of the initial 515,476 records for California newborns whose screening data had accession dates (date the specimen arrived at the laboratory) from January to December 1998, 6,325 (1.23%) could not be included because of incomplete or inadequate data. The remaining birth records totaling 509,151 in the final data file included 201 diagnosed with PCH and 989 infants with TSH level > 25 μU/mL. Of the 509,151 California newborns with complete data, 342,257 newborns were identified as residing in communities where groundwater wells were tested for ClO_4_^−^. Of these records, 50,326 newborns from 24 communities resided in areas with average ClO_4_^−^ concentrations in drinking water sources > 5 μg/L, and 291,931 newborns from 287 communities resided in areas with average ClO_4_^−^ concentrations ≤5 μg/L. No apparent biases were evident as a result of the 3,584 records deleted because of various missing demographic data or screening test characteristics. Overall, 0.95% of records were deleted from the communities with average ClO_4_^−^ concentrations > 5 μg/L compared with 1.05% deleted from communities with average ClO_4_^−^ concentrations ≤5 μg/L in drinking water (Appendix 1). In addition, a sensitivity analysis, in which newborns from cities and towns where groundwater was not tested were classified into the low concentration category, did not significantly change the study findings.

### DWP water survey.

Overall, communities from 20 California counties were tested for ClO_4_^−^ (Appendix 2). Four southern California counties (Los Angeles, Orange, Riverside, and San Bernardino) and one northern California county (Sacramento) had a total of 24 communities with average ClO_4_^−^ concentrations in drinking water > 5 μg/L, based on the California DWP data for 1997–1998. In the same five counties, there were 199 communities with average ClO_4_^−^ concentrations ≤5 μg/L.

### Primary congenital hypothyroidism.

Of the 201 newborns diagnosed with PCH in California in 1998, 141 newborns were from communities receiving drinking water that had been tested for ClO_4_^−^ (Table 1). Fifteen cases (10.6%) were from areas with average ClO_4_^−^ concentrations in drinking water > 5 μg/L (20.4 cases were expected), and 126 (89.4%) cases of PCH were from communities with average concentrations of ClO_4_^−^ in drinking water ≤5 μg/L (Table 1). After controlling for sex, ethnicity, multiple birth status, and birth weight, the POR for PCH was not increased for 1998 California newborns whose mothers resided in communities with average ClO_4_^−^ concentrations > 5 μg/L (POR = 0.71; 95% CI, 0.40–1.19) (Table 2). For newborns in the normal birth weight category (2,500–4,000 g), the POR was slightly lower for newborns from communities with average ClO_4_^−^ concentration > 5 μg/L (POR = 0.64; 95% CI, 0.32–1.15) than the results for all birth weights (Table 2).

Among newborns from areas that did not receive Colorado River water but had average ClO_4_^−^ concentrations > 5 μg/L, the POR for PCH was near 1 (POR = 1.14; 95% CI, 0.52–2.28). In this analysis of communities that did not receive Colorado River water, the OR for newborns in the normal birth weight category (POR = 1.01; 95% CI, 0.39–2.26) was slightly lower than the OR for all newborns. The POR among communities that received Colorado River water was not elevated (POR = 0.43; 95% CI, 0.15–0.96).

Similar to previous reports of California newborn data (Lorey and Cunningham 1992; Waller et al. 2000), female newborns had a higher risk for PCH (POR = 1.94; 95% CI, 1.37–2.78). Variation by race/ethnicity status was also observed with Asians (POR = 1.83; 95% CI, 0.94–3.50) and Hispanics (POR = 2.17; 95% CI, 1.36–3.63) having higher risks, and African Americans (POR = 0.29; 95% CI, 0.05–0.99) having lower risks compared with whites. Low birth weight status (< 2,500 grams) was also associated with PCH (POR = 1.90; 95% CI, 0.99–3.31).

### TSH.

There were 684 newborns identified as having high TSH levels among 342,257 newborns screened from communities where drinking water was tested for ClO_4_^−^ (Table 1). Of these, 537 (78.5%) were from communities with average ClO_4_^−^ concentrations in drinking water ≤5 μg/L, and 147 (21.5%) were from areas with average ClO_4_^−^ concentrations > 5 μg/L.

**Figure 1:**
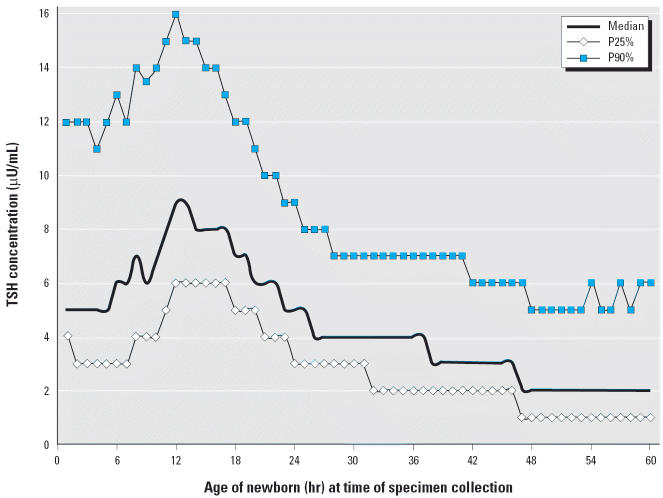
TSH concentrations at 25th, median, and 90th percentiles by specimen collection time, California newborns, 1998. Included only newborns from areas where water system groundwater was tested for ClO_4_^−^ in 1997–1998.

TSH concentrations rose rapidly as expected in the first 12 hr of life, then declined, stabilized by 24 hr after birth, and continued to decline (Figure 1). Among newborns from communities with drinking water tested for ClO_4_^−^, 123,583 (36.1%) had their blood sample collected for TSH screening at < 24 hr of age. The majority of newborns (79.7%) with high TSH in both exposure groups were screened at < 24 hr of age (Table 3). Of the 102,966 newborns from communities that did not receive Colorado River water and had water system groundwater wells tested for ClO_4_^−^, 230 newborns were identified as having high TSH, and 188 (81.7%) of these newborns had blood specimens collected < 24 hr (Table 3).

The adjusted POR associated with high TSH among newborns screened ≥24 hr of age and whose mothers resided in communities with average ClO_4_^−^ concentrations > 5 μg/L was 0.73 (95% CI, 0.40–1.23) (Table 4). For newborns of normal birth weight, screened ≥24 hr of age, the OR for high TSH was also not elevated (POR = 0.74; 95% CI, 0.37–1.33).

For communities that did not receive Colorado River water, the POR for high TSH followed a similar pattern. The adjusted POR for high TSH in communities with average ClO_4_^−^ concentrations > 5 μg/L was 0.87 (95% CI, 0.37–1.83). Among normal birth weight newborns, the POR for high TSH was also not increased (POR = 0.71; 95% CI, 0.24–1.77) (Table 4). Among southern California residents who received Colorado River water, the POR was not elevated (POR = 0.57; 95% CI, 0.22–1.20) (Table 4).

Of the demographic factors and birth characteristics examined in our multivariate models, females had a higher risk for high TSH (POR = 1.89; 95% CI, 1.33–2.71) compared with males. By race/ethnicity status, results for high TSH were similar to the findings for PCH, with modest elevations for Asians (POR = 1.37; 95% CI, 0.73–2.48) and Hispanics (POR = 1.40; 95% CI, 0.91–2.20) and a decrease for African Americans (POR = 0.40; 95% CI, 0.12–1.02) compared with whites. Birth weight and multiple birth status were not associated with high TSH (data not shown).

## Discussion

This statewide study was initiated as a follow-up analysis to a previous study of a southern California community (Kelsh et al. 2003) to verify whether similar findings would be observed in a larger study population. In addition to the increased sample size, this study of 1998 California NBS Program data offered several other analytical advantages. First, 1998 was the first year that TSH testing was conducted for all California newborns. Second, ClO_4_^−^ drinking water monitoring data for the period 1997–1998 were available for a large number of drinking water sources in California to link with the NBS Program data. We observed 15 cases of PCH in 1998 in areas of California where ClO_4_^−^ was detected at average concentrations > 5 μg/L, while 20.4 cases were expected. When we compared PCH cases in communities with average ClO_4_^−^ concentration > 5 μg/L that did not receive Colorado River water, we did not find an excess number of cases, nor was an excess number of cases observed among the population receiving Colorado River water as a drinking source.

In addition, we did not find evidence of high TSH levels for California newborns whose mothers resided in communities with average ClO_4_^−^ drinking water concentrations > 5 μg/L. When we compared California communities that did not receive Colorado River water but had average ClO_4_^−^ concentrations > 5 μg/L, there was also no excess risk of high TSH levels.

Although slightly more than one third of all newborns in the study population were screened within the first 24 hr of life, the majority of newborns (79.7%) with high TSH concentrations were screened within the first day. The strong effect of time of sample collection on TSH level dictated our focus on TSH results after 24 hr of age. Given the large number of newborns with high TSH screened within the first 24 hr of life, this approach was considered more appropriate than analyses including all newborns regardless of sampling age and attempting to control for this powerful confounding factor in the data analysis.

### Limitations.

The California DHS uses nine screening laboratories that serve various geographic regions of the state. Thus, it is possible that laboratory variation may affect the outcome of TSH screening as a result of slight variations in methods and procedures and changes in personnel. This possibility was evaluated and appears unlikely, because the GDB uses standardized protocols and rigorous quality control procedures for all laboratory procedures. In addition, laboratory variation would more likely occur across years rather than within 1 year, as we have analyzed here.

Because we were using data for drinking water at a community level, the exposure classification protocol that we adopted does not account for variation due to individual consumption patterns (e.g., bottled water) or migration into and out of communities resulting from residential mobility or travel to work sites outside of mothers’ residential communities. In addition, the very dynamic and complex water systems for many of the study communities rely on several water sources, which often contribute different proportions of water at different times of the year. Specific water allocation data were not available for many of the different water companies.

Our method of averaging ClO_4_^−^ concentrations based on water testing assumed that each water source contributed an equal proportion of water to the communities it served. Because of the uncertainty of this assumption, we used this calculation only to group communities into categories of potentially exposed (estimated average ClO_4_^−^concentration > 5 μg/L) and likely not exposed (estimated average ClO_4_^−^concentration ≤ 5 μg/L). This method does not incorporate personal water consumption patterns or the mixing of multiple water sources by water companies. Given the limitation of this method, these calculations were not used to conduct potential dose–response analyses.

### Comparison with previous epidemiologic studies of newborns.

Previous published studies have evaluated potential associations between T_4_, TSH, or PCH and ClO_4_^−^ exposure in newborn populations in California, Nevada, Arizona, and Chile (Brechner et al. 2000; Crump et al. 2000; Kelsh et al. 2003; Lamm and Doemland 1999; Li et al. 2000a, 2000b). We focused our analysis on PCH as a significant disease and on TSH as a sensitive indicator of thyroid function. TSH is considered a more sensitive and specific indicator for assessing subclinical thyroid function than T_4_ (Nordyke et al. 1998) and is the preferred screening test for PCH (American Academy of Pediatrics 1993).

The results of the current study were similar to those of previous studies with respect to risk factors for PCH and elevated TSH levels (Kelsh et al. 2003; Lorey and Cunningham 1992; Waller et al. 2000). We found that ethnicity, sex, and birth weight were consistent predictors of PCH, whereas specimen collection time had the most pronounced effect on TSH levels (Allen et al. 1990; Waller et al. 2000). Blood samples collected at < 24 hr of age had much higher TSH levels than those collected 1 day or more after birth. When age at specimen collection time was taken into account, no differences in TSH level were observed between newborns from communities with average ClO_4_^−^ concentrations in drinking water ≤ 5 μg/L and > 5 μg/L.

In this analysis of California newborns, we studied a much larger number of newborns with TSH results compared with previous studies in Chile, Arizona, and Nevada (Brechner et al. 2000; Crump et al. 2000; Li et al. 2000a). However, in each of those smaller studies, exposure information was likely more precise because the communities studied relied solely on one water supply, whereas across California, communities often rely on multiple water sources.

Our findings are consistent with previously published analyses of exposure to ClO_4_^−^ in drinking water and PCH and TSH, with the exception of the analysis of Arizona newborns by Brechner et al. (2000). No increase in the number of PCH cases was found in counties where ClO_4_^−^ had been detected in California and Nevada well water supplies (Lamm and Doemland 1999). Likewise, there was no association between TSH concentration and low-level ClO_4_^−^ exposures in drinking water among newborns in a study of Las Vegas and Reno, Nevada (Li et al. 2000a). In a Chilean study of three regions with high, medium, and nondetectable ClO_4_^−^ concentrations in drinking water (Crump et al. 2000), no associations between newborns’ TSH concentrations and ClO_4_^−^ region or between childhood TSH concentrations and lifetime residence in the three regions were reported. Our results were also similar to the findings of a 15-year study of newborns in a southern California community (Kelsh et al. 2003). However, our results differed from those reported among Arizona newborns (Brechner et al. 2000). Further investigation of this study population suggests that differences in medical procedures, hospital practices, or other regional or demographic factors provide a more likely explanation of the TSH concentration differences observed for newborns in Yuma and Flagstaff than drinking water exposure to ClO_4_^−^ (Goodman 2001; Lamm 2003).

Despite use of the same California newborn screening data (although for different years), our results do not support the findings of an unpublished analysis of ClO_4_^−^ and TSH of California newborns in 1996, when the two-stage screening for PCH was in place (Schwartz 2001). As previously described, methodological and analytic differences between the study presented here and that of Schwartz do not allow for direct comparison of findings (Kelsh et al. 2003). These differences include evaluation of TSH data as a continuous variable in the Schwartz analyses and a dichotomous variable in this study. Analysis of TSH as a continuous variable would have to address the issue that more than 50% of the newborns screened had a value of 5 μU/mL recorded, because any TSH concentration below the limit of detection was recorded or censored at 5 μU/mL. Although this truncation of the data would not pose a problem for a screening program, ignoring the censoring of the data would distort the results when they are analyzed as a continuous variable in analysis of variance models. In addition, results reported by Schwartz suggest that several variables, especially the newborn blood specimen collection hour, appear to have been processed incorrectly in the Schwartz analyses, leading to misclassification of a significant number of newborn data records. The exposure assignment procedures used by Schwartz were also different but were not adequately documented to permit a comparison with our protocol. Thus, the different time periods examined, the different statistical analyses applied, the different exposure assignment procedures implemented, and the data misclassification errors that we identified in the Schwartz analyses have led to different results and conclusions between the current investigation and the Schwartz unpublished analysis.

### Other studies.

In addition to the community studies of newborn thyroid function and ClO_4_^−^ concentrations in drinking water, several adult studies of thyroid disease and cancer have not identified an association with ClO_4_^−^ in drinking water or occupational ClO_4_^−^ exposure (Gibbs et al. 1998; Lamm et al. 1999; Li et al. 2001; Morgan and Cassady 2002).

In an early study of treatment for hyper-thyroidism, 11 of 12 newborns of mothers taking potassium ClO_4_^−^ at 600 mg or 1,000 mg daily exhibited no abnormalities, while one had a transiently enlarged thyroid (Crooks and Wayne 1960). A recent volunteer study suggested that ClO_4_^−^ in drinking water at the equivalent of 180–220 μg/L daily results in no effect on thyroid function (Greer et al. 2002). Given that the ClO_4_^−^ concentrations in California communities’ drinking water, where ClO_4_^−^ was detected, had a median value of 12 μg/L with a range from 5 to 87 μg/L, most of the testing results were below the detection limit. These concentrations, along with the results of the studies by Crooks and Wayne (1960) and Greer et al. (2002), argue against adverse health effects among California newborns.

## Conclusions

In the current study of potential ClO_4_^−^ exposure in drinking water and PCH and high TSH concentrations, we found that PCH rates in California communities where ClO_4_^−^ concentrations in drinking water average > 5 μg/L were equal to or lower than those in communities with average ClO_4_^−^ concentrations ≤ 5 μg/L. We also found no statistically or biologically relevant differences between newborns in these communities with respect to TSH concentrations. These findings are consistent with the medical literature, which reports that most cases of PCH result from developmental defects of the thyroid. In addition, most epidemiologic studies to date have not associated PCH or newborn TSH levels with exposures to ClO_4_^−^ in drinking water. Recently, the National Academies’ National Research Council Committee to Assess the Health Implications of Perchlorate Ingestion (2005) reviewed the available data from animal, human, and epidemiologic studies, with greater emphasis placed on controlled human studies such as Greer et al. (2002). The committee concluded that the data across animal, human volunteer, and epidemiologic studies were not consistent with a causal association between exposure to ClO_4_^−^ in the drinking water and either congenital hypothyroidism or thyroid function in normal full-term newborns (National Research Council 2005). Our findings in this epidemiologic study were consistent with that conclusion. Despite the limitations of using aggregate-level exposure data and the relatively small number of cases of PCH and high TSH levels even in this full statewide analysis, these results suggest that exposure to ClO_4_^−^ in drinking water supplies in California at the levels reported does not appear to be associated with either PCH or high TSH levels.

## Figures and Tables

**Table 1:** Number of newborns tested, number with high TSH, and number of PCH cases by ClO_4_^−^ exposure classification,^a^ California, 1998.

	Population after data editing^b^		
Exposure classification	Total	High TSH (%)	PCH (%)
All newborns from California communities tested for ClO_4_^−^	342,257	684 (0.20)	141 (0.04)
Newborns from communities with average ClO_4_^−^ concentrations > 5 μg/L	50,326	147 (0.29)	15 (0.03)
Newborns from communities with average ClO_4_^−^ concentrations ≤ 5 μg/L	291,931	537 (0.18)	126 (0.04)

a Testing results provided by California DHS DWP.

b Some records may have invalid or missing information for more than one variable.

**Table 2:** ORs and 95% CIs for PCH by ClO_4_^−^ exposure classification, Colorado River water status, and birth weight, California newborns,^a^ 1998.

	Average ClO_4_^−^ concentrations				
	≤ 5 μg/L		> 5 μg/L		
	No PCH	PCH	No PCH	PCH	POR^b^ (95% CI)
All California communities					
All birth weights	287,754	122	49,622	15	0.71 (0.40–1.19)
Normal birth weight^c^	241,275	100	41,615	11	0.64 (0.32–1.15)
California communities not receiving Colorado River water					
All birth weights	76,972	30	24,378	10	1.14 (0.52–2.28)
Normal birth weight	64,323	24	20,235	7	1.01 (0.39–2.26)
Colorado River water communities (California)					
All birth weights	210,782	92	25,244	5	0.43 (0.15–0.96)
Normal birth weight	176,952	76	21,380	4	0.49 (0.13–0.98)

a Only first born of multiple births were included in these analyses.

b POR was adjusted for race, sex, birth weight, multiple birth, and Colorado River water use.

c Newborns with normal birth weight (2,500–4,000 g).

**Table 3:** Distribution of high TSH [no. (%)] by ClO_4_^−^ exposure classification, Colorado River water status, and specimen collection time, California newborns, 1998.

	High TSH [*n* (%)]		
	Average ClO_4_^−^ concentrations		
	≤ 5 μg/L	> 5 μg/L	All communities tested for ClO_4_^−^
California communities			
Specimen collection < 24 hr	413 (76.9)	132 (89.8)	545 (79.7)
Specimen collection ≥ 24 hr	124 (23.1)	15 (10.2)	139 (20.3)
Total	537 (100)	147 (100)	684 (100)
California communities not receiving Colorado River water			
Specimen collection < 24 hr	124 (79.0)	64 (87.7)	188 (81.7)
Specimen collection ≥24 hr	33 (21.0)	9 (12.3)	42 (18.3)
Total	157 (100)	73 (100)	230 (100)
Colorado River water communities (California)			
Specimen collection < 24 hr	289 (76.1)	68 (91.9)	357 (78.6)
Specimen collection ≥24 hr	91 (23.9)	6 (8.1)	97 (21.4)
Total	380 (100)	74 (100)	545 (100)

**Table 4:** ORs and 95% CIs for high TSH by ClO_4_^−^ exposure classification, Colorado River water status, and birth weight, California newborns,^a^ 1998.

	Average ClO_4_^−^ concentrations				
	≤5 μg/L		> 5 μg/L		
	Normal TSH	High TSH	Normal TSH	High TSH	POR^b^ (95% CI)
All California communities					
All birth weights	185,409	119	29,100	14	0.73 (0.40–1.23)
Normal birth weight^c^	152,266	94	23,679	11	0.74 (0.37–1.33)
California communities not receiving Colorado River water					
All birth weights	46,653	32	13,113	8	0.87 (0.37–1.83)
Normal birth weight	38,121	24	10,462	5	0.71 (0.24–1.77)
Colorado River water communities (California)					
All birth weights	138,756	87	15,987	6	0.57 (0.22–1.20)
Normal birth weight	114,145	70	13,217	6	0.70 (0.27–1.49)

a Multiple births represented once; specimen collection ≥24 hr for all newborns was analyzed and adjusted for ethnicity, sex, multiple birth status, and birth weight.

b POR was adjusted for race, sex, birth weight, multiple birth, and Colorado River water use.

c Newborns with normal birth weight (2,500–4,000 g).

## Appendix 1

**Appendix 1:** Summary of data editing, cleaning, and imputation^a^ applied to California newborn screening data, 1998.

	Newborns from communities with average ClO_4_^−^ concentrations					
	> 5 μg/L		≤ 5 μg/L		Newborns from communities tested for ClO_4_^−^	
	Newborn	Cases	Newborn	Cases	Newborn	Cases
Initial data	50,811	15	295,030	127	345,841	142
Imputed or corrected	*n* (%)	*n*	*n* (%)	*n*	*n* (%)	*n*
Collection age imputed^b^	317 (0.6)	0	3,044 (1.0)	1	3,361 (1.0)	1
Date of birth corrected	40 (0.1)	0	369 (0.1)	0	409 (0.1)	0
Ethnic status assigned to Unknown^c^	592 (1.2)	0	2,809 (1.0)	3	3,401 (1.0)	3
Deleted						
Multiple records	72 (0.1)	0	356 (0.1)	0	428 (0.1)	0
Birth weight^d^	383 (0.8)	0	2,450 (0.8)	1	2,833 (0.8)	1
Sex	35 (0.1)	0	316 (0.1)	0	351 (0.1)	0
Out-of-state mother	0 (—)	0	0 (—)	0	0 (—)	0
Other hypothyroidism^e^	1 (< 0.1)	0	6 (< 0.1)	0	7 (< 0.1)	0
TSH, TSH determination	0 (—)	0	1 (< 0.1)	0	1 (< 0.1)	0
Total records deleted	485 (1.0)	0	3,099 (1.1)	1	3,584 (1.0)	1
Total	50,326	15	291,931	126	342,257	141

a Some records may have invalid or missing information for more than one variable.

b Total of 5,144 newborns with missing collection age were recovered; 1,951 were imputed by drawing randomly from a normal distribution.

c Missing ethnic status assigned to Unknown category.

d Total of 4,685 newborns with missing birth weights, 56 with weights < 250 g and 72 with weights > 7,500 g.

e Information on other forms of hypothyroidism is not routinely reported because the California DHS considers these data unreliable and not appropriate for analytical purposes.

## Appendix 2

**Appendix 2:** Counties with water systems tested for ClO_4_^−^, California, 1998.

	No. of communities with ClO_4_^−^ concentration		
County	≤ 5 μg/L	> 5 μg/L	Communities > 5 μg/L
Alameda	4	0	
Butte	1	0	
Contra Costa	1	0	
Inyo	3	0	
Kern	5	0	
Los Angeles	130	12	Azusa, Baldwin Park, Claremont, Covina,Glendora, Hacienda Heights, Industry,Irwindale, La Mirada, La Puente, La Verne, Newhall
Monterey	1	0	
Orange	32	1	La Habra
Riverside	21	1	Riverside
Sacramento	2	3	Gold River, Rancho Cordova,
Sacramento
San Bernardino	14	7	Bloomington, Chino, Colton, Fontana, Redlands, Rialto, San Bernardino
San Diego	28	0	
San Francisco	1	0	
San Mateo	16	0	
Santa Barbara	2	0	
Santa Clara	16	0	
Solano	1	0	
Sutter	1	0	
Ventura	7	0	
Yuba	1	0	
Total	287	24	
